# Chemopreventive activity of GEN-27, a genistein derivative, in colitis-associated cancer is mediated by p65-CDX2-β-catenin axis

**DOI:** 10.18632/oncotarget.7554

**Published:** 2016-02-21

**Authors:** Qianming Du, Yajing Wang, Chao Liu, Hong Wang, Huimin Fan, Yan Li, Jianing Wang, Xu Zhang, Jinrong Lu, Hui Ji, Rong Hu

**Affiliations:** ^1^ State Key Laboratory of Natural Medicines, Department of Physiology, China Pharmaceutical University, Jiangsu, Nanjing, P.R. China; ^2^ Department of Chronic Communicable Disease, Jiangsu Provincial Center for Disease Prevention and Control, Jiangsu, Nanjing, P.R.China; ^3^ Neurobiology Laboratory, Jiangsu Center for Drug Screening, China Pharmaceutical University, Jiangsu, Nanjing, P.R.China; ^4^ College of Clinical Medicine, Chengdu University of TCM, Chengdu, P.R. China; ^5^ Department of Organic Chemistry, China Pharmaceutical University, Jiangsu, Nanjing, P.R. China

**Keywords:** genistein-27, colitis-associated cancer, CDX2, β-catenin, chemoprevention

## Abstract

Nonresolving inflammation in the intestine predisposes individuals to colitis-associated colorectal cancer (CAC), which leads to high morbidity and mortality. Here we show that genistein-27 (GEN-27), a derivative of genistein, inhibited proliferation of human colorectal cancer cells through inhibiting β-catenin activity. Our results showed that GEN-27 increased expressions of adenomatous polyposis coli (APC) and axis inhibition protein 2 (AXIN2), and reduced β-catenin nuclear localization, which resulted from the inhibition of NF-κB/p65 nuclear localization and up-regulation of caudal-related homeobox transcription factor 2 (CDX2). Furthermore, GEN-27 decreased binding of p65 to the silencer region of CDX2 and increased binding of CDX2 to the promoter regions of APC and AXIN2, thus inhibiting the activation of β-catenin induced by TNF-α. Importantly, GEN-27 protected mice from azoxymethane (AOM)/dextran sodium sulfate (DSS)-induced colon carcinogenesis, with reduced mortality, tumor number and tumor volume. Histopathology, immunohistochemistry and flow cytometry revealed that dietary GEN-27 significantly decreased secretion of proinflammatory cytokines and macrophage infiltration. Moreover, GEN-27 inhibited AOM/DSS-induced p65 and β-catenin nuclear translocation, while promoted the expression of CDX2, APC, and AXIN2. Taken together, our findings demonstrate that the anti-proliferation effect of GEN-27 *in vitro* and the prevention of CAC *in vivo* is mediated by p65-CDX2-β-catenin axis via inhibiting β-catenin target genes. Our results imply that GEN-27 could be a promising candidate for the chemoprevention of CAC.

## INTRODUCTION

Colorectal cancer (CRC) is the third most common malignant neoplasm worldwide and the second leading cause of cancer related deaths in the United States [1]. As the seventh hallmark of cancer, cancer-associated inflammation is closely related to the development of colorectal cancer along with other factors such as epigenetic abnormalities and genetic mutations [2-4]. It has been shown that patients suffering from inflammatory bowel diseases (IBD), including ulcerative colitis and Crohn disease, are at increased risk of developing CRC [5-7]. Therefore, anti-inflammatory and anti-carcinogenic chemopreventive interventions for the high-risk individuals are considered a viable alternative among experts. Owing to the availability and safety, dietary supplements have become a viable and important strategy for preventing CRC [1].

Deregulation or constitutive activation of the Wnt/β-catenin pathway has been shown to contribute to the initiation and progression of different kinds of human disorders, including early stages of sporadic CRC and IBD-associated carcinogenesis [8, 9]. In normal cells, most of β-catenin binds to cadherin in the cytomembrane, regulating cell-cell adhesion, while β-catenin in the cytoplasm is rapidly phosphorylated by glycogen synthase kinase-3 beta (GSK3β) at Ser33, Ser37, and Thr41 in the adenomatous polyposis coli (APC), axis inhibition protein 2 (AXIN2), GSK-3β destruction complex and is subsequently degraded by the proteasome [10]. In tumor cells, inactivation of the APC/AXIN/GSK-3β complex causes β-catenin accumulation in the cytosol and its translocation into the nucleus, where it acts as a co-activator for TCF/LEF-mediated transcription and modulates cell proliferation, survival and differentiation [11]. This destruction complex thereby controls the proliferation of colon cells by maintaining the level of active β-catenin.

It has been shown that innate immune cells in the stroma, such as tumor-associated macrophages, play a crucial role in tumor growth, invasion and neovascularization [12, 13]. Tumor necrosis factor-α (TNF-α), for example, is a proinflammatory cytokine secreted by macrophages in acute inflammatory response [14], and during sustained inflammation TNF-α level within colonic mucosa is increased, which contributes to the invasiveness of adenocarcinomas [15]. Due to the ability of inducing growth factors and suppressing apoptosis, TNF-α or activation of NF-κB pathway is required for the progression of hepatocellular carcinoma [16] and colorectal cancers [17, 18]. Furthermore, TNF-α has been reported to increase the activity of Wnt/β-catenin pathway in gastric tumor cells, colorectal cancer cells and in a colitis-associated cancer mouse model [18-20].

The caudal-related homeobox transcription factor 2 (CDX2) is an important transcription factor which controls the balance between cell proliferation and differentiation in intestinal epithelium [21]. It has been shown that CDX2 is required for cytodifferentiation and villus morphology of the intestinal cells [22-24]. Decreased expression of CDX2 is often observed at the invasive front and in tumor buddings [25, 26]. Owing to the low rate of mutations in the CDX2 gene, it is likely that a regulatory mechanism rather than genetic alterations contributes to the down-regulation of CDX2. Indeed, RELA/p65, the subunit of the key inflammatory transcription factor NFKB/NF-κB, is claimed one of the regulators of CDX2 in colon cancer cells [27]. Furthermore, CDX2 is able to enhance the expressions of APC and AXIN2 [28, 29], which lead to stabilization of the degradation complex of cytoplasmic β-catenin. Therefore, it is possible that the enhanced tumorigenicity of CAC is caused by the down-regulated CDX2 through activating β-catenin. Therefore, a better understanding of the mutual regulation of inflammation and carcinogenesis by TNF-α, NF-κB/p65, CDX2 and β-catenin will be essential for discovering new therapeutics for CAC.

**Figure 1:**
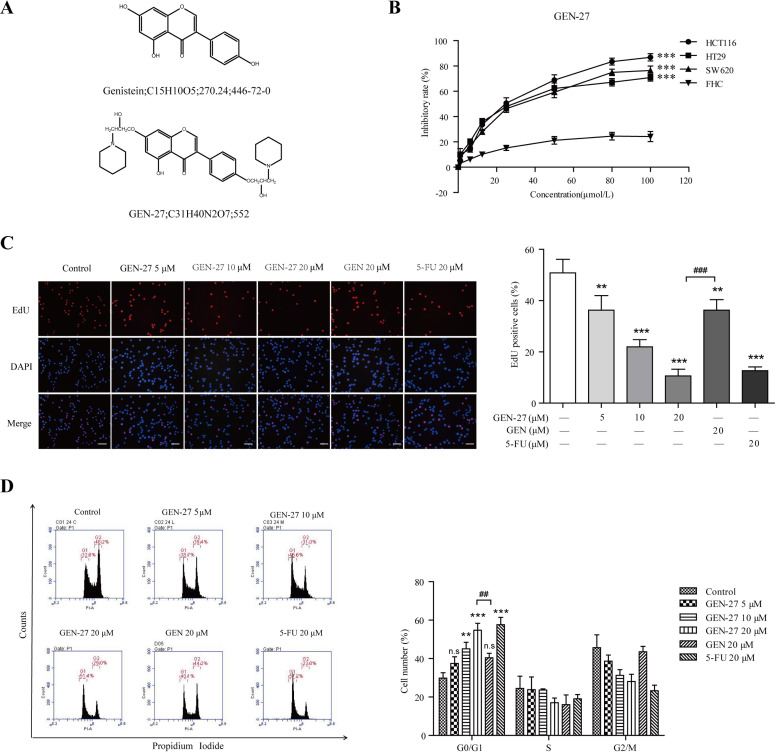
GEN-27 inhibits proliferation of human colorectal carcinoma cells **A.** The chemical structure of genistein (GEN) and genistein-27 (GEN-27). **B.** HCT116, HT29, SW620 and FHC cells were seeded into 96-well plates at a density of 5 × 10^3^ cells/well and then treated with various concentrations of GEN-27 for 24 hours and cell viability was determined using MTT assay. Values are expressed as mean ± SD (*n* = 5). **C.** HCT116 cells were treated with GEN (20 μM), 5-FU (20 μM) and the indicated concentrations of GEN-27 for 24 hours. Cell proliferation assay was measured using immunofluorescence cytochemistry (scale bar, 100 μm). The percentage of EdU positive cells are shown as the mean ± SD (*n* = 3). *P < 0.05, **P < 0.01, ***P < 0.001 with control group; ^###^P < 0.001. **D.** HCT116 cells were treated with GEN (20μM), 5-FU (20 μM) and the indicated concentrations of GEN-27 for 24 hours. Cell cycle distribution was measured using flow cytometry. The percentage of cells in each population are shown as the mean ± SD (*n* = 3). *P < 0.05, **P < 0.01, ***P < 0.001 with control group; ^##^*P* < 0.01. The data shown are representative of three experiments.

Genistein (GEN, Figure 1A) is a natural isoflavone found in soybeans, a leguminous plant popular in Asian countries. It exerts diverse biological functions including chemopreventive properties against different kinds of cancers including colorectal cancer [30, 31]. Therefore, thirty derivatives of GEN were designed and synthesized, and among them, genistein-27(GEN-27) (Figure 1A) exhibits much more potent anti-proliferation activity against colon cancer cells compared with others. Previously, GEN was reported to inhibit AOM-induced colorectal cancer [32]. However, the effect of GEN and its derivatives on colitis-associated colon tumor development has not been investigated.

In the present study, we demonstrated that GEN-27 significantly inhibited proliferation of human colorectal cancer cells through inhibiting the activity of p65-CDX2-β-catenin axis. Importantly, supplementing the diet with GEN-27 protects mice against azoxymethane (AOM)/dextran sodium sulfate (DSS)-induced colon carcinogenesis,indicating that GEN-27 could potentially be usedfor the chemoprevention of colitis-associated cancer.

## RESULTS

### GEN-27 inhibits proliferation of human colorectal carcinoma cells

The chemical structures of genistein (GEN) and genistein-27 (GEN-27) were shown in Figure 1A. In order to access the effect of GEN-27 in human relevant models, human colorectal carcinoma cell lines HCT116, HT29, SW620 and normal colon epithelial cell line FHC were treated with varying concentrations of GEN-27 for 24h (Figure 1B). A marked anti-proliferative activity was observed in HCT116, HT29 and SW620 cells with IC50 value of 23.64 μM, 28.96 μM and 30.43 μM, respectively. However, GEN-27 showed a lower cytotoxicity in normal colon FHC cells, which indicates that cancer cells are more sensitive to GEN-27 than normal cells. As shown in Figure 1C, the percentage EdU-positive HCT116 cells were reduced by GEN-27 in a dose-dependent manner, which confirmed the anti-proliferation effect of GEN-27. Cell cycle distribution of HCT116 cells was examined and a dose-dependent G0/G1 phase arrest induced by GEN-27 (5, 10, 20 μM) was observed (Figure 1D). Altogether, these data demonstrate that GEN-27 inhibits colon cancer cell proliferation *in vitro* through blocking cell cycle progression.

### GEN-27 inhibits β-catenin activity in human colorectal tumor cells

**Figure 2:**
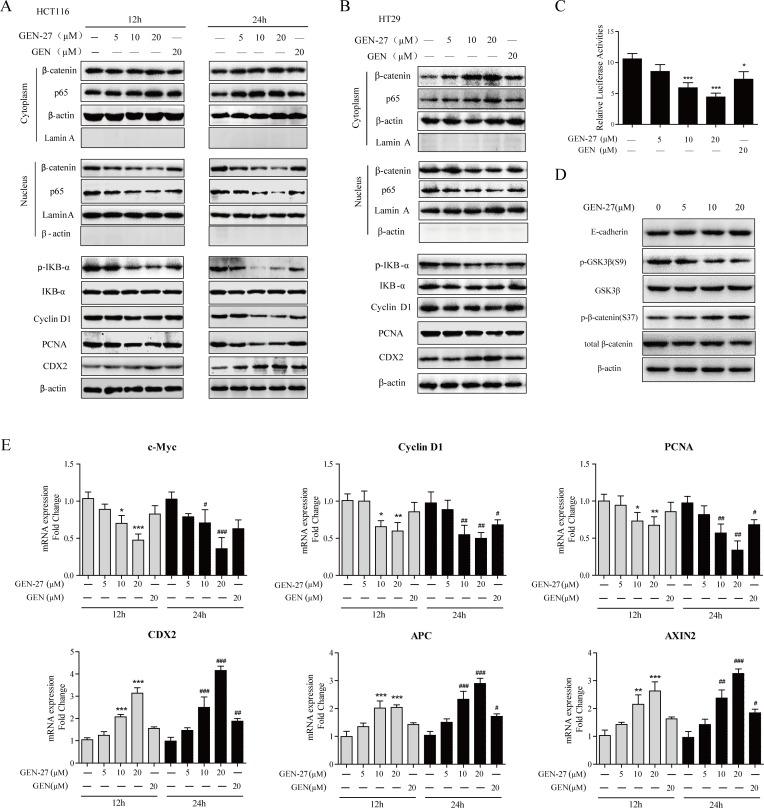
GEN-27 inhibits β-catenin activity in human colorectal tumor cells **A.**, **B.**, **D.** NF-κB/p65, β-catenin nuclear translocation and protein levels of p-IκB-α, IκB-α, Cyclin D1, PCNA, CDX2, E-cadherin, p-GSK3β (S9), GSK3β, p-β-catenin (S37) and total β-catenin were determined by western blot. Data shown are representative of 3 experiments. **C.** Effects of the indicated concentrations of GEN-27 and GEN (20μM) on β-catenin transcriptional activity of HCT116 cells. TOP- or FOP-flash luciferase plasmid was co-transfected into HCT116 with renilla luciferase plasmid. Luciferase activity was divided by renilla activity. Results are quoted as relative values *vs* the control value and plotted as mean ± SD (*n* = 3). **E.** The mRNA expressions of c-Myc, Cyclin D1, PCNA, CDX2, APC and AXIN2 in HCT116 cells from each group were determined by Real-time PCR. Values are expressed as mean ± SD (*n* = 5). *P < 0.05, **P < 0.01, ***P < 0.001 *vs*. control group at 12h, ^#^*P* < 0.05, ^##^*P* < 0.01, ^###^*P* < 0.001 *vs*. control group at 24h. GEN, genistein; GEN-27, genistein-27.

To investigate if β-catenin is involved in the anti-proliferation effect of GEN-27, β-catenin activity was analyzed in HCT116 cells using the TOP/FOP-flash reporter system. As shown in Figure 2C, the basal activity of β-catenin was dose-dependently decreased by GEN-27. Moreover, GEN-27 time- and dose-dependently inhibited nuclear translocation of β-catenin and the protein expressions of target genes including PCNA and Cyclin D1 in HCT116 and HT29 cells (Figure 2A and 2B). Furthermore, the mRNA expressions of c-Myc, Cyclin D1 and PCNA were also decreased by GEN-27 treatment (Figure 2E). Interestingly, the mRNA and protein levels of CDX2 and the mRNA expressions of APC and AXIN2, which negatively regulate β-catenin activity, were strongly increased (Figure 2A, 2B and 2E). Given that E-cadherin and GSK3β play pivotal roles in the distribution of β-catenin, we investigated the protein levels of E-cadherin and p-GSK3β. As shown in Figure 2D, GEN-27 significantly inhibited p-GSK3β (S9) levels, without affecting total GSK3β and E-cadherin. Moreover, GEN-27 dose-dependently increased the level of p-β-catenin (Ser37), which leads to its degradation by proteasome. Altogether, these data indicate that GEN-27 inhibits the activity of β-catenin through increasing APC and AXIN2 expressions of the destruction complex and reducing the level of p-GSK3β (S9), which contributes to the stability of the destruction complex and promotes the phosphorylation of β-catenin, leading to the down-regulation of the pro-proliferation genes.

### Regulation of p65-CDX2-β-catenin axis contributes to the inhibition of Wnt/β-catenin pathway by GEN-27 *in vitro*

**Figure 3:**
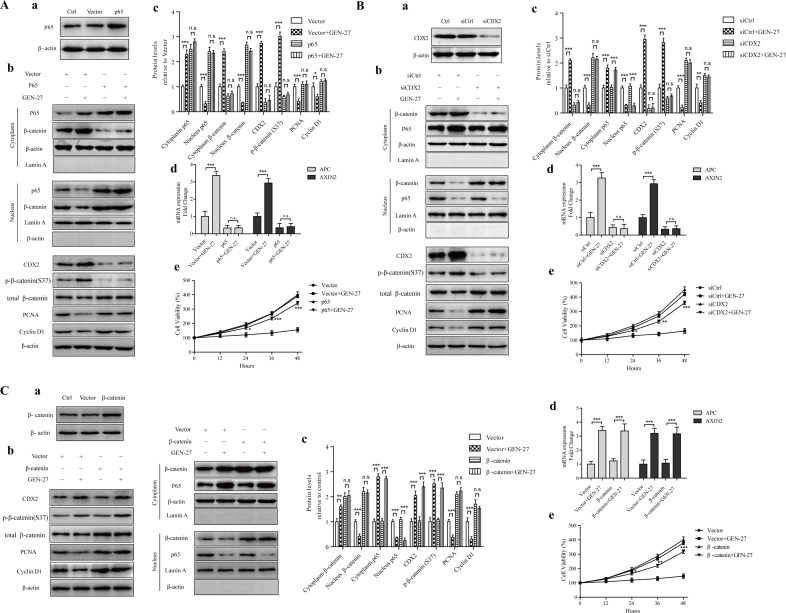
The p65-CDX2-β-catenin axis is responsible for the anti-proliferation effect of GEN-27 on HCT116 human colon cancer cells (Aa, Ba, Ca) Protein levels of p65 (Aa), CDX2 (Ba) and β-catenin (Ca) with or without p65 overexpression **A.**, CDX2 silencing **B.**, β-catenin overexpression **C.** (Ab-c, Bb-c, Cb-c) NF-κB/p65, β-catenin nuclear translocation and protein levels of CDX2, p-β-catenin(S37), total β-catenin, PCNA, and Cyclin D1 were determined by western blot. Data shown are representative of 3 experiments. *P < 0.05, **P < 0.01,***P < 0.001. (Ad, Bd, Cc) Real-time qPCR analysis of APC and AXIN2 in HCT116 cells. Values are the mean ± SD (*n* = 5). (Ae, Be, Ce) Cell viability of HCT116 human colon cancer cells at the indicated time points from each group was analyzed by MTT assay. Each point represents mean ± SD (*n* = 5). *P < 0.05, **P < 0.01,***P < 0.001 *vs*. p65+GEN-27 group (Ad), siCDX2+GEN-27 group (Bd), or β-catenin+GEN-27 group (Cd). GEN, genistein; GEN-27, genistein-27.

As GEN-27 inhibited nuclear translocation of p65 and increased the expression of CDX2 (Figure 2A and 2B), we explored if the inhibition on β-catenin nuclear translocation is related to the p65 nuclear distribution and CDX2 expression. Firstly, p65 overexpression plasmid was transfected into HCT116 cells, which was verified by Western blot (Figure 3Aa). Compared with control, the expression of CDX2, APC, AXIN2 and p-β-catenin (S37) was inhibited, while β-catenin nuclear translocation as well as its target gene expression was increased by overexpression of p65 (Figure 3Aa-d). Furthermore, the reduction of β-catenin nuclear localization and the lowered proliferation rate of HCT116 induced by GEN-27 were remarkably blocked by the increased p65 level (Figure 3Ab and e), suggesting that CDX2 and β-catenin are downstream targets of p65. Secondly, to determine if the inactivation of β-catenin induced by GEN-27 is CDX2 dependent, we knocked down CDX2 with siRNA (Figure 3Ba). As shown in Figure 3B, knock-down of the CDX2 gene significantly reversed the GEN-27-induced increase of APC, AXIN2 and p-β-catenin (S37), decrease of β-catenin nuclear translocation, down-regulation of β-catenin target genes (Figure 3Bb and d), as well as the inhibition of cell viability (Figure 3Be), while p65 nuclear translocation was not affected by the siCDX2 (Figure 3Bb), which confirmed the vital role of p65-CDX2-β-catenin axis in the anti-proliferation effect of GEN-27. Finally, to further investigate whether β-catenin has an effect on the nuclear localization of p65 and CDX2 expression, β-catenin overexpression plasmid was transfected into HCT116 cells (Figure 3Ca). It was found that the GEN-27-mediated inhibition of cell viability and the decreased protein levels of β-catenin target genes were reversed by the β-catenin overexpression, while the p65 nuclear translocation and CDX2, APC and AXIN2 expressions were not affected (Figure 3Cb-e). These observations show that GEN-27 inhibits p65 nuclear translocation, which leads to the increased expression of CDX2, APC, AXIN2 and p-β-catenin (S37), decreased nuclear translocation of β-catenin, resulting in the down-regulation of β-catenin target genes. In conclusion, GEN-27 inhibits the Wnt/β-catenin pathway through inhibiting the activity of p65-CDX2-β-catenin axis in HCT116 cells.

### GEN-27 inhibits TNF-α-induced proliferation of human colon cancer cells

**Figure 4:**
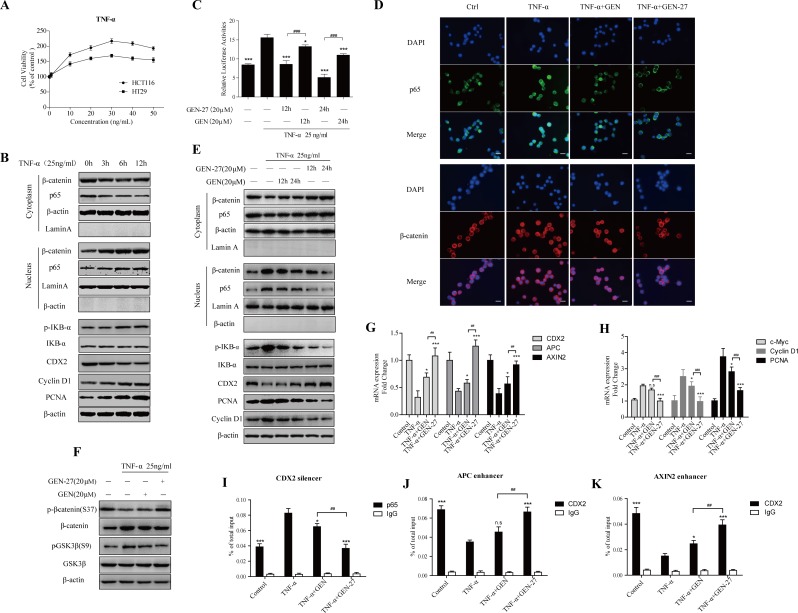
GEN-27 inhibits TNF-α-induced proliferation of human colon cancer cells **A.** Effect of TNF-α on cell proliferation of HCT116 and HT29 human colon cancer cells. Cells were treated with various concentrations (1-50 ng/ml) of TNF-α for 24 hours, and cell viability was determined using MTT assay. Values were expressed as mean ± SD (*n* = 5). **B.** HCT116 cells were treated with 25 ng/ml TNF-α for the indicated times. Proteins were collected and NF-κB/p65, β-catenin nuclear translocation and protein levels of p-IκB-α, IκB-α, CDX2, Cyclin D1 and PCNA were determined by western blot. *P < 0.05, **P < 0.01,***P < 0.001 *vs*. control group. **C.** HCT116 cells were pre-treated with GEN-27 (20μM) and GEN (20 μM) for the indicated hours, and the effect of TNF-α (25 ng/ml, 6h) on β-catenin transcriptional activity was determined using TOP- or FOP-flash TOP/FOP-flash reporter system. Results are quoted as relative values *vs* the control value and plotted as mean ± SD (*n* = 3). **D.** HCT116 cells were incubated with GEN-27 (20 μM) and GEN (20 μM) for the 24 hours, and then treated with 25ng/ml TNF-α for 6 hours. NF-kB/p65 and β-catenin nuclear translocation were analyzed by immunofluorescence cytochemistry (scale bar, 25 μm). **E.**, **F.** NF-κB/p65, β-catenin nuclear translocation and protein levels of p-IκB-α, IκB-α, CyclinD1, PCNA, CDX2, p-β-catenin (S37) and total β-catenin were determined by western blot. *P < 0.05, **P < 0.01,***P < 0.001 *vs*. the TNF-α group in (E); *P < 0.05, **P < 0.01,***P < 0.001 in (F). **G.**, **H.** The mRNA expressions of CDX2, APC, AXIN2, c-Myc, Cyclin D1 and PCNA in HCT116 cells from each group were determined by Real-time PCR. Values are expressed as mean ± SD (*n* = 5). **I.**-**K.** Chromatin immunoprecipitation (ChIP) using a p65 antibody (I) or CDX2 antibody (J and K) and negative control IgG antibody in HCT116 cells treated with the indicated factors. Immunoprecipitates were probed with primer pairs located within the CDX2 silencer (I), APC enhancer (J) or AXIN2 (K) enhancer region and analyzed by Real-time PCR. Values are shown as percentage of total input DNA and are represented as mean ± SD (*n* = 3). *P < 0.05, **P < 0.01,***P < 0.001 *vs*. the TNF-α group; ^#^*P* < 0.05, ^##^*P* < 0.01, ^###^*P* < 0.001. GEN, genistein; GEN-27, genistein-27.

Proinflammatory cytokines, such as TNF-α secreted by macrophage, promotes tumorgenesis [18-20]. TNF-α treatment caused elevated cell proliferation of HCT116 and HT29 cells as demonstrated by cell viability assay (Figure 4A), and the p65-CDX2-β-catenin axis was also activated in a time-dependent manner in HCT116 cells (Figure 4B). Compared with GEN, GEN-27 pretreatment significantly prevented TNF-α-induced activation of the p65-CDX2-β-catenin axis, which can be concluded from the β-catenin activity assay using TOP/FOP-flash reporter system (Figure 4C), immunofluorescence (Figure 4D), Western blot (Figure 4E) and Real-time PCR analysis (Figure 4G and 4H). Knock-down of CDX2 with siCDX2 abolishes GEN-27's inhibitory effect on β-catenin activity in the presence of TNF-α (Figure S1A). Moreover, the increase of p-GSK3β and the decrease of β-catenin phosphorylation at Ser37 induced by TNF-α were also reversed by pretreatment of GEN-27 (Figure 4F). It has been reported that p65 could down-regulates CDX2 expression by binding to its non-coding region [27], and CDX2 could bind to upstream enhancer elements in the APC and AXIN2 genes and therefore increase their protein levels [33]. ChIP assay was performed to demonstrate the binding of p65 on the silencer region of the CDX2 and CDX2 on the enhancer elements of APC and AXIN2. Chromatin-protein complexes from treated HCT116 cells were immunoprecipitated with a p65 (Figure 4I) or CDX2-specific antibody (Figure 4J and 4K) and an IgG antibody (negative control). Significant amplification of CDX2 silencer region after anti-p65 ChIP (Figure 4I) and reduction of APC and AXIN2 enhancer regions after anti-CDX2 ChIP (Figure 4J and 4K) were observed in the TNF-α treated group, and GEN-27 and GEN pre-treatment blocked the effect of TNF-α, which were correlated with the mRNA analysis of CDX2, APC and AXIN2 (Figure 4G). Taken together, these results indicate that GEN-27 inhibits TNF-α-induced proliferation of human colon cancer cells through down-regulating the activity of p65-CDX2-β-catenin axis and reducing the expressions of the β-catenin target genes.

### GEN-27 prevents colitis-associated tumorigenesis

**Figure 5:**
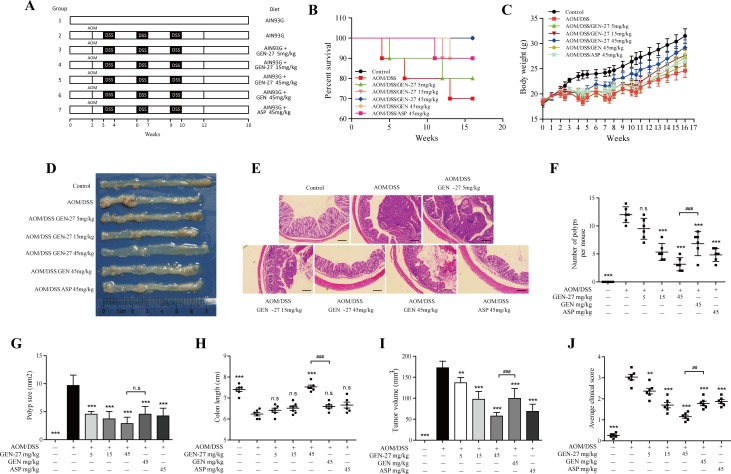
GEN-27 prevents colitis-associated tumorigenesis **A.** Induction procedure and groups designed for the AOM/DSS model of CAC (*n* = 10). **B.** Survival rate. **C.** Body weight. **D.** Representative images. **E.** H&E staining (scale bar, 100 μm). **F.** Number of polyps per mouse. **G.** Polyp size. **H.** Colon length. **I.** The tumor volume was determined by totaling the volume of all tumors for a given animal. **J.** Average clinical score of colons. Clinical parameters (weight loss, stool consistency, bleeding) of indicated mice. Values are mean ± SD (*n* = 6). *P < 0.05,**P < 0.01,***P < 0.001 *vs*. AOM+DSS group. ^#^*P* < 0.05, ^##^*P* < 0.01, ^###^*P* < 0.001. GEN, genistein; GEN-27, genistein-27; ASP, aspirin.

**Figure 6:**
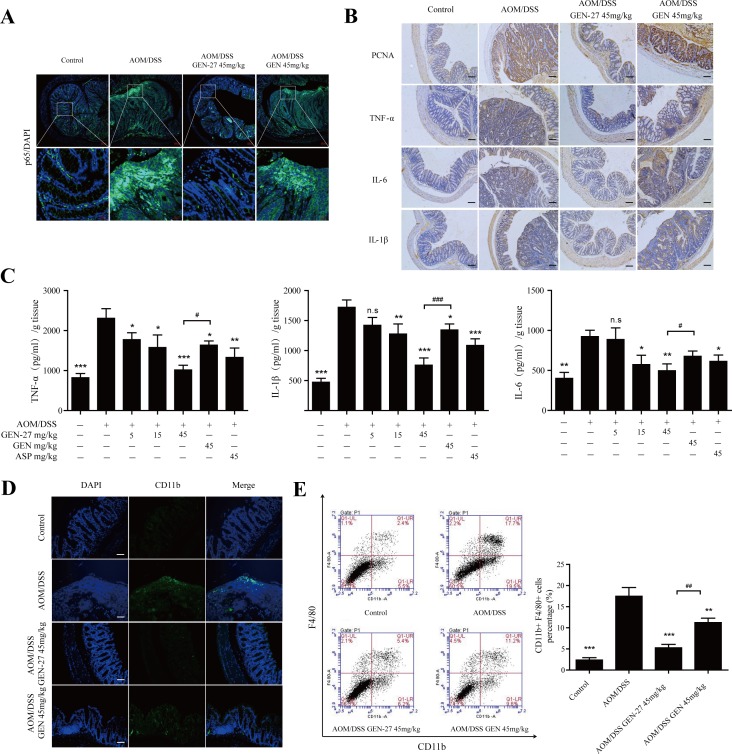
GEN-27 attenuates inflammation in the colitis-associated colorectal cancer model **A.** Expression of NF-κB/p65 was analyzed by immunofluorescence cytochemistry (scale bar, 100 μm). **B.** Protein expressions of PCNA, TNF-α, IL-6 and IL-1β were detected by immunohistochemistry (scale bar, 100 μm). **C.** Protein levels of the inflammatory cytokines TNF-α, IL-1β, and IL-6 in colon explant cultures were determined by ELISA. **D.** Sections of colonic tissue were immunostained with DAPI (blue) and anti-CD11b-FITC (green) (scale bar, 100 μm). **E.** Percentage of F4/80+ CD11b+ macrophages in infiltrated leukocytes of colon tissue. The distribution of CD11b+F4/80+ monocyte/macrophages in colonic tissues was detected by representative FACS blots. Data shown in (A, B, D and E) are representative of 3 experiments. Values are mean ± SD (*n* = 3). *P < 0.05, **P < 0.01,***P < 0.001*vs*. AOM+DSS group. ^#^ *P* < 0.05, ^##^*P* < 0.01. GEN, genistein; GEN-27, genistein-27.

To determine whether GEN-27 directly inhibits the formation of CAC, we generated an AOM/DSS induced colitis-associated colon cancer model. GEN-27 was well tolerated in mice, and no obvious systemic toxicity was observed during the entire period of treatment as indicated by the body weight, general appearance and organ histology. Based on Kaplan-Meier survival curves (Figure 5B), GEN-27, GEN and aspirin (ASP) treatment increased the survival of mice. Body weight of the mice was monitored throughout the experiment (Figure 5C). Substantial weight loss following each exposure to DSS and subsequent weight regain when being maintained on water was observed (Figure 5C). In groups of GEN-27, GEN and aspirin (ASP) treatment, the mice had a reduced weight loss and recovered more quickly than mice in the AOM/DSS group. The tumor incidence was 100% in all mice. However, fewer and smaller tumors were observed in the GEN-27 (45mg/kg) -treated group (*P* < 0.001) (Figure 5D-5G). Tumor volume and average clinical score were also reduced by GEN-27 in a dose-dependent manner, which represent the total volume of all tumors of a mouse and clinical parameters including weight loss, stool consistency and bleeding, respectively. (Figure 5I and 5J). Compared with the AOM/DSS group, there was a significant increase in colon length of mice in the GEN-27 (45mg/kg) group (*P* < 0.001) (Figure 5H). Moreover, GEN-27 treatment inhibited the PCNA expression, which indicates the cell proliferation in the colon (Figure 6B). Hence, the data obtained here strongly suggest that a diet supplemented with GEN-27 prevents colorectal tumorigenesis in a mouse model of colitis associated CRC.

### GEN-27 attenuates inflammation in a colitis-associated colorectal cancer model

In addition to the decreased colitis-associated tumorigenesis in AOM/DSS-treated mice, we found that the inflammation level was markedly reduced by GEN-27. Nuclear translocation of NF-κB/p65 was markedly reduced by GEN-27 compared with the AOM/DSS group as shown by immunofluorescence cytochemistry (Figure 6A). Expressions of proinflammatory cytokines TNF-α, IL-6 and IL-1β were also significantly suppressed (Figure 6B and 6C). Besides, we examined the macrophage infiltration in colon tissues. Fewer CD11b+ cells from GEN-27(45mg/kg)-treated mice were detected compared with those from AOM/DSS group (Figure 6D). The accumulation of macrophages in the colons was also investigated with flow cytometry (Figure 6E). The percentage of F4/80+/CD11b+ cells increased from 2.4% to 17.7% in colon tissue from AOM/DSS group, and it reduced to 5.4% and 11.2% after GEN-27 and GEN treatment, respectively. This suppressive effect of GEN-27 was correlated with its inhibition of colonic inflammatory cytokines (Figure 6B and 6C). Taken together, these results indicate that GEN-27 treatment reduces macrophage activity and thereby ameliorates AOM/DSS induced inflammation in a colitis-associated colorectal cancer model.

### GEN-27 inhibits the p65-CDX2-β-catenin axis in the AOM/DSS induced colitis-associated colorectal cancer model

**Figure 7:**
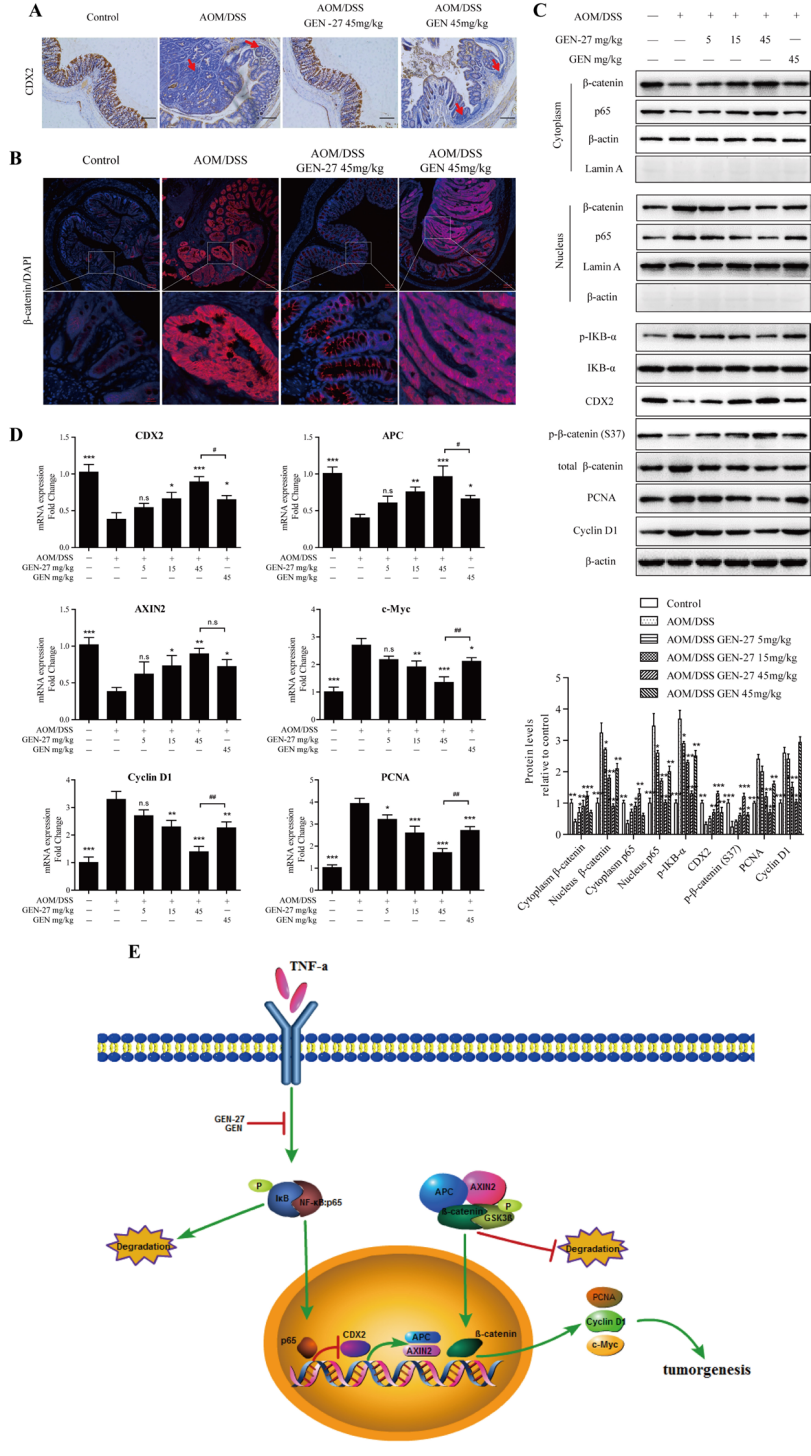
GEN-27 inhibits the Wnt/β-catenin pathway in a AOM/DSS induced colorectal cancer model **A.** The expression of CDX2 was detected by immunohistochemistry. Red arrow, cells with CDX2 loss. Data shown are representative of 3 experiments. **B.** Expression of β-catenin were analyzed by immunofluorescence cytochemistry. **C.** NF-κB/p65, β-catenin nuclear translocation and protein levels of p-IκB-α, IκB-α, Cyclin D1, PCNA, CDX2, p-β-catenin (S37) and total β-catenin were determined by western blot. Data shown are representative of 3 experiments. *P < 0.05, **P < 0.01,***P< 0.001 *vs*. AOM+DSS group. **D.** The mRNA expressions of CDX2, APC, AXIN2, Cyclin D1, c-Myc and PCNA in colon sections were determined by real-time PCR. Values are mean ± SD (*n* = 5). **E.** Schematic diagram depicting the role of GEN-27 on Wnt/β-catenin pathway. GEN-27 inhibits TNF-α-induced phosphorylation of IκB-α, and prevents the nuclear translocation of p65, which increases the protein expression of CDX2. And the up-regulated CDX2 increases APC and AXIN2 gene expression, which activates the destruction complex and promotes the phosphorylation of β-catenin at Ser37. As a consequence, GEN-27 inhibits the target gene expressions including PCNA, Cyclin D1 and c-Myc, prevents the colitis-associated initiation, promotion, and progression of tumor development. *P < 0.05, **P < 0.01, ***P < 0.001 *vs*. AOM+DSS group. # *P* < 0.05, ## *P* < 0.01. GEN, genistein; GEN-27, genistein-27.

In order to determine if the inactivation of p65-CDX2-β-catenin axis is responsible for the chemoprevention effect of GEN-27 *in vivo*, the related protein and mRNA levels from mouse colons were assessed. As expected, the activity of p65-CDX2-β-catenin axis was markedly inhibited by GEN-27 compared with the AOM/DSS group. Nuclear translocation of p65 (Figure 6A and Figure 7C) and β-catenin (Figure 7B and 7C), protein expressions of p-IKB-α, PCNA and Cyclin D1 (Figure 7C), and mRNA expressions of c-Myc, Cyclin D1 and PCNA (Figure 7D) in colonic tissue were remarkably down-regulated. Besides, GEN-27 diet deeply increased the protein and mRNA expression of the CDX2 and the mRNA levels of APC and AXIN2 compared with the AOM/DSS group, which contributes to the phosphorylation and degradation of β-catenin (Figure 7A, 7C and 7D). Together, these results indicate that GEN-27 administration prevent colitis-associated tumorigenesis through inhibiting the activity of the p65-CDX2-β-catenin axis *in vivo*.

## DISCUSSION

It has been reported that genistein (GEN) inhibits AOM-induced colorectal cancer [30, 32]. Our results also confirmed this inhibitory effect of GEN on Wnt/β-catenin pathway in mice with CAC (Figure 7B-7D) and in HCT116 cells with or without TNF-α stimulation (Figure 2A-2E and Figure 4C-4K). Compared with its parent compound, GEN-27 exhibits much more potent activities of chemoprevention against CAC in mice and anti-proliferation against human colorectal cancer cells. Furthermore, the present data highlight the role of CDX2 in the NFκB/p65-promoted activation of β-catenin signaling. In the process of CAC, inflammatory cytokines, such as TNF-α, induce phosphorylation of IκB-α, and promote the nuclear translocation of p65, which inhibits the protein expression of CDX2. And the down-regulated CDX2 decreases APC and AXIN2 gene expression, which inactivates the destruction complex and inhibits the phosphorylation of β-catenin at Ser37. As a consequence, TNF-α induces the β-catenin target gene expressions including PCNA, Cyclin D1 and c-Myc, thus promoting the colitis-associated tumor development.

Dietary GEN-27 reduced inflammation, neutrophil infiltration, cell proliferation, tumor burden, and mortality associated with the AOM/DSS treatment. NF-κB/p65 and β-catenin nuclear localization induced by AOM/DSS were also reduced in the GEN-27-fed mice and in HCT116 and HT29 colon cancer cells treated with GEN-27. GEN-27 did not affect the expression E-cadherin, but reduced the level of p-GSK3β(S9), which results in the increase of p-β-catenin (S37) (Figure 2D). These results suggest that the anti-inflammatory and anti-cancer activity of GEN-27 is related to the activation of the destruction complex, which phosphorylates β-catenin at Ser37 leading to its destruction by the proteasome.

The proinflammatory cytokine TNF-α is a critical mediator of inflammatory disorders and is likely to activate signaling pathways involved in tumorigenesis [17, 18]. And Wnt/β-catenin pathway can be activated by TNF-α in gastric tumor cells, colorectal cancer cells, as well as in a colitis-associated cancer mouse model [18-20]. We demonstrate that TNF-α at the concentrations of 0-50 ng/ml promotes proliferation of HCT116 and HT29 cells through increasing β-catenin activity (Figure 4A and 4B). Besides, in AOM/DSS-induced CAC mice model, the protein level of TNF-α is markedly elevated (Figure 6B and 6C), which indicates the connection between inflammation and cancer. GEN-27 time-dependently inhibited TNF-α-induced activation of β-catenin *in vitro* and dose-dependently reduce TNF-α secretion *in vivo* (Figures 4 and 6), which accounts for its anti-proliferation and anti-inflammation effects.

It has been recently demonstrated that CDX2 regulates gene expression of APC and AXIN2, components of the β-catenin degradation complex [33], and increasing evidences indicate that CDX2 inhibits the activity of β-catenin and thereby the progression of the colorectal cancer [34, 35]. Previous studies reported that CDX2 loss could be observed at the invasive front [20, 33], while we detected CDX2 loss not only at the invasive front but also in the adenoma induced by AOM/DSS in mice (Figure 7A, red arrow). This is not surprising, because the protein level of TNF-α is greatly elevated owing to induction of DSS in the CAC model (Figure 6B and 6C), which deeply inhibits the expression of CDX2 in tumor cells and malignant transformed cells at the invasive front.

A few number of studies have focused on the activity and regulation of CDX2 expression. The published works indicate that extracellular signal-regulated kinase 1/2 (ERK1/2) or p38 MAPK may be regulators of CDX2 [36-39]. It is demonstrated that TNF-α regulates the CDX2 expression through NF-κB in HT29 cells [27]. In our study, we demonstrate that p65, as an up-steam regulator, down-regulates CDX2 expression through binding to the silencer region of the CDX2, and inhibiting the mRNA expression of CDX2 at transcriptional level. Furthermore, by using gain- and loss- of- function approaches, our results clearly indicate that GEN-27 inhibits cancer cell proliferation and inactivates Wnt pathway through regulating the p65-CDX2-β-catenin axis, and this is the first report that p65 could increase the activity of β-catenin through down-regulating CDX2 expression at the transcriptional level.

In conclusion, our study provides strong evidence for the chemopreventive activity of GEN-27 against colitis-associated colorectal cancer in mice through inhibiting Wnt/β-catenin pathway. The mechanism involves p-IκB-α-p65 pathway mediated CDX2-dependent activation of the destruction complex, thus leading to decreased expressions of β-catenin target genes including cyclin D1, PCNA and c-Myc. GEN-27 could potentially be used for the chemoprevention of colitis-associated cancer.

## MATERIALS AND METHODS

### Cell culture

Human colon cancer cell lines (HCT116, HT29, SW620) and normal colon epithelial FHC cell line were obtained from Cell Bank of the Chinese Academic of Sciences (Shanghai, China) and cultured in McCoy's 5a medium supplemented with 10% FBS, RPMI-1640 medium supplemented with 10% FBS, DMEM medium containing 10% FBS and DMEM medium containing 10% FBS, respectively, in a 37°C humidified incubator with 5% CO2. GEN-27 was synthesized in Organic Chemistry Laboratory of China Pharmaceutical University (>98% purity, Nanjing Jiangsu, China). GEN and Fluorouracil (5-FU) were purchased from Sigma-Aldrich (>98% purity, St. Louis, MO). Cells were treated with GEN-27, GEN or 5-FU at indicated concentrations and time points, followed by TNF-α (Peprotech) based on pre-determined experimental design. Cell viability was assessed by MTT assay.

### EdU cell proliferation assay

HCT116 cells were seeded in 24-well plates at a density of 2 × 105 cells per well. After adhesion, cells were cultured in a standard culture medium or treated with GEN-27 (5, 10, 20 μM), GEN (20 μM), and 5-FU (20 μM) for 24 h. Cell proliferation was evaluated by measuring EdU incorporation during DNA synthesis according to the manufacturer's instructions (Baseclick). EdU incorporation was measured using immunofluorescence.

### Cell cycle assay

After treatment with GEN-27 (5, 10, 20 μM), GEN (20 μM), and 5-FU (20 μM) for 24 h, HCT116 cells were harvested, spin down and the resulting pellets were fixed in ice-cold 70% ethanol. Fixed cells were centrifuged, washed and re-suspended in PBS containing RNase A (1 mg/ml), and propidium iodide (PI) was added (1.0 mg/ml). PI-stained cells were analyzed by a fluorescence-activated cell sorter (Accuri^®^ C6, Becton Dickinson, Franklin Lakes, NJ, USA), followed by the determination of the percentage of cells in G0/G1, S, and G2/M.

### TOP and FOP flash reporter assays

For the analysis of β-catenin transcriptional activity, HCT116 cells were co-transfected with TOP (T-cell factor reporter plasmid)- or FOP (mutant T-cell factor reporter plasmid)-flash luciferase and renilla luciferase plasmid. Luciferase activity was divided by renilla activity to normalize transfection efficiency.

### Western blot

The protocols for western blot have been reported previously [40]. Proteins were extracted in lysis buffer (30 mM Tris, pH 7.5, 150 mM sodium chloride, 1 mM phenylmethylsulfonyl fluoride, 1 mM sodium orthovanadate, 1% Nonidet P-40,10% glycerol, and phosphatase and protease inhibitors), separated by SDS-PAGE and electrophcoretically transferred onto polyvinylidene fluoride membranes (Roche Applied Science). The membranes were probed with antibodies overnight at 4°C, and then incubated with a horse radish peroxidase-coupled secondary antibody. Detection was performed using a Tanon chemiluminescent substrate system. The cell lysates were immunoblotted using the following primary antibodies: anti-β-catenin (cat. no. ab32572) (Abcam); anti-NF-κB/P65 (cat. no. 6956S), anti-IκB-α (cat. no. 9242), anti-p-IκB-α (Ser32) (cat. no. 2859) (Cell Signaling Technology); and anti-cyclin D1 (H-295) (cat. no. sc-753) (Santa Cruz Biotechnology); anti-GSK3β (cat. no. BS1402), anti-p-GSK3β (S9) (BS4084), anti-p-β-catenin (S37) (cat. no. BS4739), anti-CDX2 (cat. no. MB0125), anti-PCNA (cat. no. BS6438), anti-Lamin A (cat. no. BS1446), anti-β-actin (D8) (cat. no. AP0731) (Bioworld Technology). All primary antibodies were used at 1:1,000. Secondary antibodies include the following: goat anti-rabbit HRP (cat. no.Ab203-01), goat anti-mouse HRP (cat. no. Ab201-01) (Vazyme Biotech); all secondary antibodies were used at 1:40,000.

### Real-time PCR

RNA samples were reverse transcribed to cDNA and the PCR reactions were performed using TaKaRa SYBR Green Master Mix (Code. no. 638320) carried out in StepOnePlus™ Real-Time PCR instrument (cat. no. 4376600, Life Technologies). The program for amplification was 1 cycle of 95°C for 2 min followed by 40 cycles of 95°C for 10 s, 60°C for 30 s, and 95°C for 10 s. The primer sequences used in this study were listed in the Supplementary data. The PCR results were normalized to Gapdh expression and were quantified by the ΔΔCT method.

### Immunofluorescence

The immunofluorescence assay for NF-kB/p65 and β-catenin nuclear translocation was performed according to the method previously described [41].

### Small interfering RNAs, plasmids, and transfection

NF-kB/p65 plasmid and β-catenin plasmid (Addgene, Cambridge, MA, USA) and CDX2 siRNA (Santa Cruz Biotechnology, Danvers, MA, USA) transfections were performed according to the manufacturer's instructions of ExFect^TM^ Transfection Reagent (Vazyme Biotech). The extent of gene knockdown and overexpression was determined by western blot.

### Chromatin immune-precipitation (ChIP) assay

The HCT116 cells were cross-linked and sonicated according to the protocol published by Nelson et al [42]. Briefly, immunoprecipitation was performed in four replicates and carried out at 4°C overnight with 1μg of rabbit anti-human p65 antibody (Cell Signaling Technology) or rabbit anti-human CDX2 antibody (BioGenex Laboratories) or an irrelevant IgG antibody as a negative control. Immunocomplexes were recovered with 50 μl protein A/G beads (Beyotime Biotechnology). Input DNA and purified immunoprecipitated DNA were analyzed by Real-time PCR. The primers used to amplify the genomic sequences of CDX2 at P65 target loci and AXIN2, APC at CDX2 target loci were listed in the Supplementary data. Quantification of the ChIP-DNA was performed using the method described by Frank et al [43].

### Animal studies and colitis-associated colon cancer

Animal welfare and experimental procedures were performed in accordance with the Guide for the Care and Use of Laboratory Animals (National Institutes of Health, the United States) and the related ethical regulations of our university. Pathogen-free male C57BL/6 mice were purchased from the Model Animal Research Center of Nanjing University (Nanjing, China) at 5 weeks of age and were exposed to a 12:12-hour light/dark cycle. Mice were given an AIN-93G purified diet (Normal diet) from Xietong-organism Co., LLC (Nanjing Jiangsu, China) and drinking water *ad libitum*. At 6 weeks of age (Week 0, Figure 5A), mice were started on the indicated diet (Supplementary Table 2) till the end of the experiment, and two weeks later, mice were injected intraperitoneally with AOM (Sigma, St. Louis, MO) at a dose of 10 mg/kg body weight, followed by 3 cycles of 2.5% DSS (MP Biomedicals LL, Solon, OH) given in drinking water for a week and then switch back to normal drinking water for 2 weeks. The body weights were measured during the experiment. Aspirin were obtained from Sigma-Aldrich Co., LLC (>98% purity, St. Louis, MO). Purified diets were prepared by Xietong-organism Co., LLC (Nanjing Jiangsu, China). Mice were allowed to eat ad libitum. Mice were euthanized at the end of the experiment and colon tissue harvested.

Preparation of colonic cell suspension and flow cytometry were performed as previously described [44].

Body weight, the presence of rectal bleeding, and stool consistency were scored and averaged to generate a semiquantative clinical score as previously described [45]. In brief, body weight, colon lengths and stool consistency were assessed at the completion of study. Rectal bleeding was assessed by sampling for the presence of blood in the stool using a Hemoccult Immunochemical Fecal Occult Blood Test (Abon Biopharm CO., LTD) as previously described [46].

### Histopathology

Colon tissues were collected and fixed in 4% formaldehyde overnight and stored in 70% ethanol. The fixed portion of the colon tissue was embedded in paraffin, cut into 6-μm part, and put onto the microscopic slides. Slides were either stained with hematoxylin-eosin (H&E) for histological analysis by optical microscopy or stained by immunohistochemistry for the proliferation marker PCNA, IL-1β, IL-6, TNF-α (Bioworld Technology), β-catenin (Abcam), p65 (Cell Signaling Technology), and counterstained using 3,3′-diaminobenzidine (DAB) followed by hematoxylin counterstain. Histopathology analysis was performed as previously described [47].

### Cytokine analysis by ELISA

Colon inflammatory cytokine was assessed by flushing mice colons with PBS, and the distal-most 1-cm^2^ colon sections were cultured in RPMI media containing penicillin/streptomycin for 24 h. Supernatants from these cultures were removed, cleared of debris by centrifugation and assessed for mouse IL-6 (EK0411), TNF-α (EK0527), IL-1β (EK0394) (Boster Biological Technology) by ELISA.

### Statistical analysis

Most results are presented as the mean ± SD. Significance between two groups was assessed by the Student's two-tailed t-test. The non-parametric Mann-Whitney U-test was used to assess significance between two means of data sets lacking a normal distribution and having a small sample size. Data sets consisting of more than 2 groups were analyzed by analysis of variance (ANOVA) with Tukey-Kramer HSD post test for multiple comparisons if significance was determined. The product limit method of Kaplan and Meier was used for generating the survival curves. P value that was less than 0.05 was considered statistically significant for all data sets. All statistical analysis was performed using GraphPad Prism software.

## SUPPLEMENTARY MATERIAL TABLES AND FIGURE



## References

[1] Jemal A, Bray F, Center MM, Ferlay J, Ward E, Forman D (2011). Global cancer statistics. CA: a cancer journal for clinicians.

[2] Fearon ER (2011). Molecular genetics of colorectal cancer. Annual review of pathology.

[3] Rogler G Inflammatory bowel disease cancer risk, detection and surveillance. Digestive diseases.

[4] Hanahan D, Weinberg RA (2011). Hallmarks of cancer: the next generation. Cell.

[5] Eaden JA, Abrams KR, Mayberry JF (2001). The risk of colorectal cancer in ulcerative colitis: a meta-analysis. Gut.

[6] Jess T, Gamborg M, Matzen P, Munkholm P, Sorensen TI (2005). Increased risk of intestinal cancer in Crohn's disease: a meta-analysis of population-based cohort studies. The American journal of gastroenterology.

[7] Ullman TA, Itzkowitz SH (2011). Intestinal inflammation and cancer. Gastroenterology.

[8] Nelson WJ, Nusse R (2004). Convergence of Wnt, beta-catenin, and cadherin pathways. Science.

[9] Herr P, Hausmann G, Basler K (2012). WNT secretion and signalling in human disease. Trends in molecular medicine.

[10] Zeng X, Tamai K, Doble B, Li S, Huang H, Habas R, Okamura H, Woodgett J, He X (2005). A dual-kinase mechanism for Wnt co-receptor phosphorylation and activation. Nature.

[11] Serafino A, Moroni N, Zonfrillo M, Andreola F, Mercuri L, Nicotera G, Nunziata J, Ricci R, Antinori A, Rasi G, Pierimarchi P (2014). WNT-pathway components as predictive markers useful for diagnosis, prevention and therapy in inflammatory bowel disease and sporadic colorectal cancer. Oncotarget.

[12] Pollard JW (2004). Tumour-educated macrophages promote tumour progression and metastasis. Nature reviews Cancer.

[13] Etoh T, Shibuta K, Barnard GF, Kitano S, Mori M (2000). Angiogenin expression in human colorectal cancer: the role of focal macrophage infiltration. Clinical cancer research : an official journal of the American Association for Cancer Research.

[14] Cox GW, Melillo G, Chattopadhyay U, Mullet D, Fertel RH, Varesio L (1992). Tumor necrosis factor-alpha-dependent production of reactive nitrogen intermediates mediates IFN-gamma plus IL-2-induced murine macrophage tumoricidal activity. Journal of immunology.

[15] Breese EJ, Michie CA, Nicholls SW, Murch SH, Williams CB, Domizio P, Walker-Smith JA, MacDonald TT (1994). Tumor necrosis factor alpha-producing cells in the intestinal mucosa of children with inflammatory bowel disease. Gastroenterology.

[16] Pikarsky E, Porat RM, Stein I, Abramovitch R, Amit S, Kasem S, Gutkovich-Pyest E, Urieli-Shoval S, Galun E, Ben-Neriah Y (2004). NF-kappaB functions as a tumour promoter in inflammation-associated cancer. Nature.

[17] Greten FR, Eckmann L, Greten TF, Park JM, Li ZW, Egan LJ, Kagnoff MF, Karin M (2004). IKKbeta links inflammation and tumorigenesis in a mouse model of colitis-associated cancer. Cell.

[18] Popivanova BK, Kitamura K, Wu Y, Kondo T, Kagaya T, Kaneko S, Oshima M, Fujii C, Mukaida N (2008). Blocking TNF-alpha in mice reduces colorectal carcinogenesis associated with chronic colitis. The Journal of clinical investigation.

[19] Oguma K, Oshima H, Aoki M, Uchio R, Naka K, Nakamura S, Hirao A, Saya H, Taketo MM, Oshima M (2008). Activated macrophages promote Wnt signalling through tumour necrosis factor-alpha in gastric tumour cells. The EMBO journal.

[20] Coskun M, Olsen AK, Bzorek M, Holck S, Engel UH, Nielsen OH, Troelsen JT (2014). Involvement of CDX2 in the cross talk between TNF-alpha and Wnt signaling pathway in the colon cancer cell line Caco-2. Carcinogenesis.

[21] Olsen AK, Boyd M, Danielsen ET, Troelsen JT (2012). Current and emerging approaches to define intestinal epithelium-specific transcriptional networks. American journal of physiology Gastrointestinal and liver physiology.

[22] Verzi MP, Shin H, He HH, Sulahian R, Meyer CA, Montgomery RK, Fleet JC, Brown M, Liu XS, Shivdasani RA (2010). Differentiation-specific histone modifications reveal dynamic chromatin interactions and partners for the intestinal transcription factor CDX2. Developmental cell.

[23] Crissey MA, Guo RJ, Funakoshi S, Kong J, Liu J, Lynch JP (2011). Cdx2 levels modulate intestinal epithelium maturity and Paneth cell development. Gastroenterology.

[24] Gao N, White P, Kaestner KH (2009). Establishment of intestinal identity and epithelial-mesenchymal signaling by Cdx2. Developmental cell.

[25] Brabletz T, Spaderna S, Kolb J, Hlubek F, Faller G, Bruns CJ, Jung A, Nentwich J, Duluc I, Domon-Dell C, Kirchner T, Freund JN (2004). Down-regulation of the homeodomain factor Cdx2 in colorectal cancer by collagen type I: an active role for the tumor environment in malignant tumor progression. Cancer research.

[26] Gross I, Duluc I, Benameur T, Calon A, Martin E, Brabletz T, Kedinger M, Domon-Dell C, Freund JN (2008). The intestine-specific homeobox gene Cdx2 decreases mobility and antagonizes dissemination of colon cancer cells. Oncogene.

[27] Kim S, Domon-Dell C, Wang Q, Chung DH, Di Cristofano A, Pandolfi PP, Freund JN, Evers BM (2002). PTEN and TNF-alpha regulation of the intestinal-specific Cdx-2 homeobox gene through a PI3K, PKB/Akt, and NF-kappaB-dependent pathway. Gastroenterology.

[28] Clevers H (2006). Wnt/beta-catenin signaling in development and disease. Cell.

[29] Gregorieff A, Pinto D, Begthel H, Destree O, Kielman M, Clevers H (2005). Expression pattern of Wnt signaling components in the adult intestine. Gastroenterology.

[30] Zhang Y, Li Q, Zhou D, Chen H (2013). Genistein, a soya isoflavone, prevents azoxymethane-induced up-regulation of WNT/beta-catenin signalling and reduces colon pre-neoplasia in rats. The British journal of nutrition.

[31] Zhang Y, Li Q, Chen H (2013). DNA methylation and histone modifications of Wnt genes by genistein during colon cancer development. Carcinogenesis.

[32] Thiagarajan DG, Bennink MR, Bourquin LD, Kavas FA (1998). Prevention of precancerous colonic lesions in rats by soy flakes, soy flour, genistein, and calcium. The American journal of clinical nutrition.

[33] Olsen AK, Coskun M, Bzorek M, Kristensen MH, Danielsen ET, Jorgensen S, Olsen J, Engel U, Holck S, Troelsen JT (2013). Regulation of APC and AXIN2 expression by intestinal tumor suppressor CDX2 in colon cancer cells. Carcinogenesis.

[34] Hinkel I, Duluc I, Martin E, Guenot D, Freund JN, Gross I (2012). Cdx2 controls expression of the protocadherin Mucdhl, an inhibitor of growth and beta-catenin activity in colon cancer cells. Gastroenterology.

[35] Guo RJ, Funakoshi S, Lee HH, Kong J, Lynch JP (2010). The intestine-specific transcription factor Cdx2 inhibits beta-catenin/TCF transcriptional activity by disrupting the beta-catenin-TCF protein complex. Carcinogenesis.

[36] Lemieux E, Boucher MJ, Mongrain S, Boudreau F, Asselin C, Rivard N (2011). Constitutive activation of the MEK/ERK pathway inhibits intestinal epithelial cell differentiation. American journal of physiology Gastrointestinal and liver physiology.

[37] Rings EH, Boudreau F, Taylor JK, Moffett J, Suh ER, Traber PG (2001). Phosphorylation of the serine 60 residue within the Cdx2 activation domain mediates its transactivation capacity. Gastroenterology.

[38] Krueger F, Madeja Z, Hemberger M, McMahon M, Cook SJ, Gaunt SJ (2009). Down-regulation of Cdx2 in colorectal carcinoma cells by the Raf-MEK-ERK 1/2 pathway. Cellular signalling.

[39] Houde M, Laprise P, Jean D, Blais M, Asselin C, Rivard N (2001). Intestinal epithelial cell differentiation involves activation of p38 mitogen-activated protein kinase that regulates the homeobox transcription factor CDX2. The Journal of biological chemistry.

[40] Tsumagari K, Abd Elmageed ZY, Sholl AB, Friedlander P, Abdraboh M, Xing M, Boulares AH, Kandil E (2015). Simultaneous suppression of the MAP kinase and NF-kappaB pathways provides a robust therapeutic potential for thyroid cancer. Cancer letters.

[41] Yao J, Hu R, Sun J, Lin B, Zhao L, Sha Y, Zhu B, You QD, Yan T, Guo QL (2014). Oroxylin A prevents inflammation-related tumor through down-regulation of inflammatory gene expression by inhibiting NF-kappaB signaling. Molecular carcinogenesis.

[42] Nelson JD, Denisenko O, Bomsztyk K (2006). Protocol for the fast chromatin immunoprecipitation (ChIP) method. Nature protocols.

[43] Frank SR, Schroeder M, Fernandez P, Taubert S, Amati B (2001). Binding of c-Myc to chromatin mediates mitogen-induced acetylation of histone H4 and gene activation. Genes & development.

[44] Wu XF, Ouyang ZJ, Feng LL, Chen G, Guo WJ, Shen Y, Wu XD, Sun Y, Xu Q (2014). Suppression of NF-kappaB signaling and NLRP3 inflammasome activation in macrophages is responsible for the amelioration of experimental murine colitis by the natural compound fraxinellone. Toxicology and applied pharmacology.

[45] Siegmund B, Lehr HA, Fantuzzi G, Dinarello CA (2001). IL-1 beta -converting enzyme (caspase-1) in intestinal inflammation. Proceedings of the National Academy of Sciences of the United States of America.

[46] Allen IC, TeKippe EM, Woodford RM, Uronis JM, Holl EK, Rogers AB, Herfarth HH, Jobin C, Ting JP (2010). The NLRP3 inflammasome functions as a negative regulator of tumorigenesis during colitis-associated cancer. The Journal of experimental medicine.

[47] Wilson JE, Petrucelli AS, Chen L, Koblansky AA, Truax AD, Oyama Y, Rogers AB, Brickey WJ, Wang Y, Schneider M, Muhlbauer M, Chou WC, Barker BR, Jobin C, Allbritton NL, Ramsden DA (2015). Inflammasome-independent role of AIM2 in suppressing colon tumorigenesis *via* DNA-PK and Akt. Nature medicine.

