# Supplementary material for: Chemopreventive activity of GEN-27, a genistein derivative, in colitis-associated cancer is mediated by p65-CDX2-β-catenin axis
Source: Oncotarget. 2016 Feb 21;7(14):17870–84. doi: 10.18632/oncotarget.7554 (PMC4951256; doi:10.18632/oncotarget.7554)
Supplement: Supplementary file 1 [file oncotarget-07-17870-s001.pdf]

Chemopreventive activity of GEN-27, a genistein derivative, in colitis-associated cancer is mediated by p65-CDX2-β-catenin axis

Supplementary Material

Supplementary Table 1

List of primers used in this study

	Sence primer	Antisence primer
CHIP-PCR		
CDX2 silencer	TCGTTAATCACGGAAGGCCG	GGAGCAGACCTCACCATGC
APC enhancer	GTTGGTGAGGAAGGTGAAGC	CGAGAACTGAGGGTGGTACAG
AXIN2 enhancer	GGAGCAGTAAAAGGCCGTAA	CCAAACCATTGAAGCCCTTA
Real time PCR		
Mouse-Cyclin D1	CCCCTTGGGGACATGTTGTT	GCTCCCTACTCTCAGGGTGA
Mouse-PCNA	AGGGTTGGTAGTTGTCGCTG	CAAACATGGTGGCGGAGTTG
Mouse-c-Myc	CTGTGGAGAAGAGGCAAACC	TTGTGCTGGTGAGTGGAGAC
mouse-CDX2	GCAGAGCCAAGGAGAGGAA	GGAGGTCACAGGACTCAAGG
mouse-APC	TTGGACTCACCGCATCACTA	CCTGGAAAGGTCAACATCGT
mouse-AXIN2	CCATCAGCAGTGTCATACCC	CTTCGTCGTCTGTTTGGTCA
Human-Cyclin D1	CACACGGACTACAGGGGAGT	GATGGTTTCCACTTCGCAGC
Human-PCNA	CAGAGCTCTTCCCTTACGCA	GTCCTTGAGTGCCTCCAACA
Human-c-Myc	CCTCCACTCGGAAGGACTATC	TTCGCCTCTTGACATTCTCC
Human-CDX2	CAGACTACCATCCGCACCA	CATCCACTCGCACAGGTTC
Human-APC	GCTCAAACCAAGCGAGAAGT	AGCATCTGGAAGAACCTGGA
Human-AXIN2	AGAGAGACCACGCCGATTG	GGGAGGCAAGTCACCAACAT

Supplementary Table 2

Composition of diets

Formula (in g/kg)	AIN93G	GEN-27 5mg/kg	GEN-27 15mg/kg	GEN-27 45mg/kg	GEN 45mg/kg	ASP 45mg/kg
Casein	200	200	200	200	200	200
L-Cystine	300	300	300	300	300	300
Corn Starch	397.486	397.486	397.486	397.486	397.486	397.486
Maltodextrin	132	132	132	132	132	132
Sucrose	100	100	100	100	100	100
Soybean Oil	70	70	70	70	70	70
Cellulose	50	50	50	50	50	50
Mineral Mix, AIN-93G-MX（94046）	35	35	35	35	35	35
Vitamin Mix, AIN-93-VX（94047）	10	10	10	10	10	10
Choline Bitartrate	2.5	2.5	2.5	2.5	2.5	2.5
TBHQ, antioxidant	0.014	0.014	0.014	0.014	0.014	0.014
GEN-27	0	0.005	0.015	0.045	0	0
GEN	0	0	0	0	0.045	0
ASP	0	0	0	0	0	0.045

A

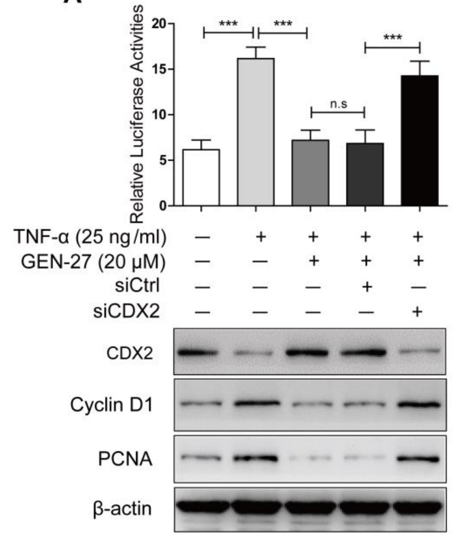

B

Regions	Sequence
CDX2 silencer region (-268 to -224)	5'-CGCACGCCAGCCTGTGGCG**GGTCTTCCCCG**CCTCTGCAGCCTAGT-3'
APC enhancer region (-572 to -528)	5'-AGGAAGGTGAAGCACT**CAGTTG**CCTTCTCGGGCCTCGGCGCCCCC-3'
AXIN2 enhancer region (-696 to -652)	5'-AGGCGGAGGGAGGAGCCGC**CATAAACTG**GAGGCAGAGTCCTTAGC-3'

NOTE. Bold letters in the CDX2 silencer region represent the NF-κB binding site and blod letters in the APC and AXIN2 enhancer region represent the CDX2 binding sites.

Figure S1. Knock-down of CDX2 abolishes GEN-27's inhibitory effect on β-catenin activity in the presence of TNF-α. (A) Effects of the indicated factors on β-catenin transcriptional activity of HCT116 cells were determined by the TOP and FOP flash reporter assay, and the protein levels of CDX2, Cyclin D1 and PCNA were analyzed by western blot. Values are expressed as mean ± s.d, and the results are representative of three independent experiments. *P < 0.05, **P < 0.01,***P<0.001. (B) The detailed sequence of CDX2 silencer region and APC, AXIN2 enhancer regions were provided.
